# Symptom Status Predicts Patient Outcomes in Persons with HIV and Comorbid Liver Disease

**DOI:** 10.1155/2012/169645

**Published:** 2012-10-03

**Authors:** Wendy A. Henderson, Angela C. Martino, Noriko Kitamura, Kevin H. Kim, Judith A. Erlen

**Affiliations:** ^1^Biobehavioral Unit, National Institute of Nursing Research, National Institutes of Health, DHHS, 10 Center Drive, Room 2-1339, Bethesda, MD 20892, USA; ^2^School of Education, University of Pittsburgh, 5918 Wesley W. Posvar Hall, 230 South Bouquet Street, Pittsburgh, PA 15261, USA; ^3^Department of Health and Community Systems, School of Nursing, University of Pittsburgh, 415 Victoria Building, 3500 Victoria Street, Pittsburgh, PA 15261, USA

## Abstract

Persons living with human immunodeficiency virus (HIV) are living longer; therefore, they are more likely to suffer significant morbidity due to potentially treatable liver diseases. Clinical evidence suggests that the growing number of individuals living with HIV and liver disease may have a poorer health-related quality of life (HRQOL) than persons living with HIV who do not have comorbid liver disease. Thus, this study examined the multiple components of HRQOL by testing Wilson and Cleary's model in a sample of 532 individuals (305 persons with HIV and 227 persons living with HIV and liver disease) using structural equation modeling. The model components include biological/physiological factors (HIV viral load, CD4 counts), symptom status (Beck Depression Inventory II and the Medical Outcomes Study HIV Health Survey (MOS-HIV) mental function), functional status (missed appointments and MOS-HIV physical function), general health perceptions (perceived burden visual analogue scale and MOS-HIV health transition), and overall quality of life (QOL) (Satisfaction with Life Scale and MOS-HIV overall QOL). The Wilson and Cleary model was found to be useful in linking clinical indicators to patient-related outcomes. The findings provide the foundation for development and future testing of targeted biobehavioral nursing interventions to improve HRQOL in persons living with HIV and liver disease.

## 1. Introduction

Since the introduction of highly active antiretroviral therapy (HAART), survival of persons living with human immunodeficiency virus (HIV) has dramatically improved. Five-year survival rate among patients following World Health Organization (WHO) standard therapies reaches approximately 75% [1]. The result is that persons living with HIV are more likely to suffer significant morbidity and mortality from other disorders such as liver disease (LD) and its related complications (anemia, end stage liver disease, lipodystrophy, and hepatocellular carcinoma) than from HIV [2]. Because of the toxic and metabolic effects of antiretroviral medications on the liver and coinfection with LD, the number of persons living with HIV and LD is increasing [3–6]. 

Hepatitis B (HBV) and C virus (HCV) infections are prevalent among HIV-infected individuals with different epidemiologic profiles, modes of transmission, natural histories, and treatments [7]. It is estimated that 3 to 6 million people are infected with HIV and chronic HBV worldwide, which is approximately 10% of HIV-positive persons. In the USA, where HBV and HIV are most often acquired by sexual transmission or injection-drug use and HBV prevalence is low, the prevalence of HIV-HBV coinfection in HIV-positive population is generally less than 10%. However, the prevalence is up to 50% among injection-drug users with HIV [8]. Generally, one-third of persons with HIV also have chronic HCV infection. In the USA, approximately 300 000 individuals are living with HIV coinfected chronic HCV [9]. HIV and HCV coinfection increases up to 70–90% of hemophilia and 60–80% of injection-drug users who have high risk of blood exposure [10].

In addition, HIV infection accelerates the natural course of HBV and HCV infections, including death, histological fibrosis/cirrhosis, decompensated liver disease, and hepatocellular carcinoma [11–13]. More recently, the evidence shows that HIV itself and immunosuppression contributes to the liver injury. Also, antiretroviral therapy (ART) attenuates the progress of HBV and HCV [12]. Furthermore, among certain populations, such as the homeless or incarcerated individuals with HIV, the prevalence of liver disease reaches 69% [14]. Nonalcoholic steatohepatitis (NASH) is also increasing in HIV-positive populations [12]. A recent study showed that fatty acid production increases in HIV and HCV coinfection [15].

Treatment advances have improved survival rates for HIV-infected individuals, although not always with a good quality of life (QOL). Deteriorated liver conditions have shown to have a significant negative effect on persons' health-related quality of life (HRQOL) [16]. In addition, HIV-positive individuals with liver disease are likely from socially vulnerable groups, such as injection-drug users, the homeless, and incarcerated people. Therefore, it is important to identify interventions that have the potential to improve QOL in people living with HIV and liver diseases (LD). An overarching goal of Healthy People 2020 is to increase life expectancy and promote QOL of individuals of all ages [17]; the challenge for researchers and practitioners is to first determine what aspects of individuals' HRQOL are affected when they live with multiple comorbid conditions.

One framework for examining HRQOL is the model proposed by Wilson and Cleary (1995) [18]. The primary aim was to test the null hypothesis, wherein, the hypothesized model would hold true with the components (biological/physiological factors, symptom status, functional status, general health perceptions, and overall quality of life) in persons living with HIV without LD and in persons living with HIV and LD. The null hypothesis was that there would be no difference in the model in persons living with HIV without LD and in persons living with HIV and LD. The secondary aim of the study was to test the relationships proposed within Wilson and Cleary's directional model of HRQOL between biological/physiological factors, symptom status, functional status, general health perceptions, and overall quality of life among persons living with HIV without LD and persons living with HIV and LD. The knowledge gained from persons with HIV and LD in this study may provide a way to support people living with this complex illness.

## 2. Materials and Methods

### 2.1. Participants

HIV-positive individuals who were currently treated with antiretroviral drugs, at least 18 years of age, and had telephone access were included in the study. All participants gave written informed consent.

### 2.2. Study Design and Data Collection

The parent study was a randomized controlled trial supported by the National Institute of Nursing Research testing interventions to improve medication adherence in persons living with HIV. Participant data was collected between April 1999 and March 2007 from western Pennsylvania and eastern Ohio community hospitals, university-based clinics, and comprehensive HIV care centers. A history of HIV and LD was ascertained by self-reported data from the Comorbidity Questionnaire, Center for Research in Chronic Disorders (CRCD), University of Pittsburgh School of Nursing (1999), and medical record review. The type of LD was confirmed through medical record review by a registered nurse and from self-report. LD included but was not limited to hepatitis (A, B, C, and other), cirrhosis, steatosis, and hepatocellular carcinoma. The precise nature of LD was not available in 5% of cases.

#### 2.2.1. Wilson and Cleary Health-Related Quality of Life Framework

HRQOL was defined as the state of perceived health and its effect on the person [18–25]. The Wilson and Cleary model of HRQOL includes health-related and social factors. As a causal model, Wilson and Cleary allows for identification of potential causal factors in the overall HRQOL paradigm.

**Figure 1:**
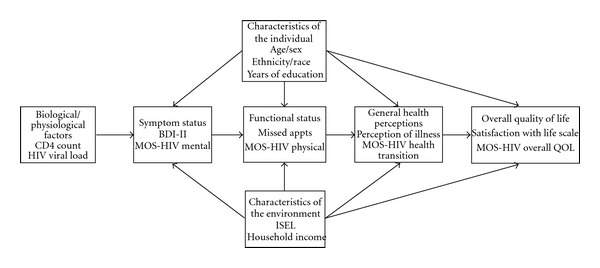
Operationalized Wilson and Cleary model, modified from [18].

Each of the components in the Wilson and Cleary model (biological/physiological factors, symptom status, functional status, general health perceptions, and overall QOL) has been addressed separately in numerous studies. The researchers reviewed those studies that were relevant to HIV and liver disease and to the operationalized Wilson and Cleary model of HRQOL (Figure 1). The definition of each parameter of Wilson and Cleary's model is described below.

**Biological/Physiological Factors**

Biological/physiological factors are any measurable function of the cells or organs of an individual. This component includes other clinical indicators such as measures of change in the function of the cell, organ, or organ system. In this study, HIV viral load and CD4 counts were used as biological and physiological factors that were assessed.

**Symptom Status**

Symptom status was described theoretically as any psychophysical, emotional, or cognitive state that influences the individual [18]. Often depressive symptoms and mental health are included in this definition. Symptom status was operationally assessed using each subject's Beck Depression Inventory II (BDI-II) score and the Medical Outcomes Study HIV Health Survey (MOS-HIV) mental function summary score [26].

**Functional Status**

Functional status was defined as one's ability to perform specific tasks such as going to work or making and keeping medical appointments [18]. Functional status was measured with the MOS-HIV physical function summary score [26] and missed clinic appointments during the prior 6 months. The MOS-HIV physical function summary score was used to report self-perceived functionality [26]. Missed clinic appointments were quantified using self-report or chart review data from the medical record review and were categorized as missed or not missed.

**General Health Perceptions**

General health perceptions were theoretically defined as how individuals perceive their own health, based on the integration of biological/physiologic factors, symptom status, and functional status combined with the effect of the particular disease or organ state on the individual [18]. General health perceptions were measured by the Perception of Illness Visual Analog Scale [27] and the single item MOS-HIV health transition score [26].

**Overall Quality of Life**

Theoretically, overall quality of life was described as how satisfied individuals are with all aspects of their life [18]. Overall quality of life was measured with the Satisfaction with Life Scale [28] and a single MOS-HIV item assessing overall HRQOL [26].

**Characteristics of the Individual**

Characteristics of the individual were specific descriptors of the person [18]. In this study, age (measured in years), sex (categorized as male or female), ethnicity/race (categorized as white and nonwhite and further categorized based on self-report), and number of years of education were included. This demographic information was collected using the CRCD Socio-demographic Questionnaire, University of Pittsburgh, School of Nursing (1999) or via medical record review.

**Characteristics of the Environment**

The characteristics of the environment theoretically incorporated all of the individual's surroundings, including tangible or intangible available resources [18]. An intangible resource that is often associated with health outcomes is social support. Social support was limited to one measure of subjective and one measure of objective social support. The subjective measure of the extent to which individuals feel that they have interpersonal resources available to them was ascertained from the total score of the Interpersonal Support Evaluation List (ISEL) [29]. Income, as a potentially tangible supportive influence from an individual's environment, was measured by annual gross household income.

### 2.3. Data Analysis

Data were compiled from the baseline data collection and medical record review from the parent study. All persons living with HIV who self-reported a “liver problem” were included in the HIV and LD group regardless of available objective medical record data. All persons living with HIV who had an LD noted in their baseline medical record were also included in the LD group. All others were considered persons living with HIV without LD.

Each variable was examined for its distribution, range, mean, median, mode, and standard deviation. Assessment was done to test for normality specific to the type of variable. For dichotomous variables, frequencies were explored to identify whether cell sizes were relatively equal. Chi-square and the independent samples *t*-test were used to examine differences between the initial and continuation studies and the two groups: persons living with HIV and LD and persons living with HIV without LD. Correlations between the variables were analyzed for systematic entry into the structured equation model (SEM). SPSS version 15.0 (SPSS Inc., Chicago, IL, USA) was used to further manage and analyze the data. 

A multigroup structural equation modeling (SEM) was used to test the fit of the Wilson and Cleary (1995) model of HRQOL in the two groups. A maximum likelihood estimation method used EQS software package version 6.1 (Multivariate Software, Inc., Encino, CA, USA) to perform the statistical analyses [30].

**Figure 2:**
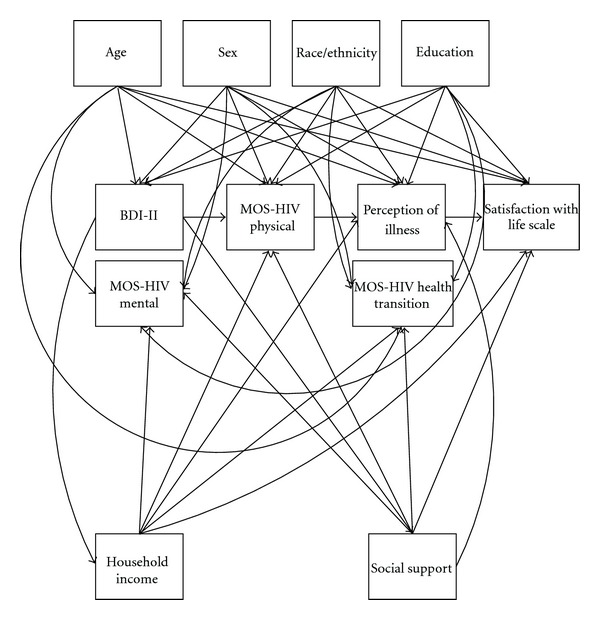
Additional 36 covariate relationships tested in exploratory SEM.

In addition, multiple pathways were added to SEM model to identify whether there were different relationships between characteristics of individuals and environment between and among persons living with HIV without liver disease and persons living with HIV and LD. Model paths included: paths from each of the four selected demographic characteristics to the four endogenous variables (symptom status, functional status, general health perceptions, and overall QOL) (16 paths); correlations among the four selected demographic characteristics (6 paths) and paths from each of the four endogenous variables to the two characteristics of the environment (household income and social support), along with the correlation between those two characteristics (9 paths). These paths are depicted graphically in Figure 2.

Age, gender, ethnicity/race, and number of years of education were included as characteristics of the individual and social support (measured with the mean score on the Inventory Support Evaluation List (ISEL)), and household income (measured as total gross annual household income) as characteristics of the environment.

For determination of adequacy of sample size the observation per parameters (N:q) was calculated. Generally, if more than five observations are present per parameter the sample size for the model is deemed adequate. In this study, the number of observations (sample size) was 532 and the number of the largest parameters included (covariates in the model) was 36. Therefore, N:q for model testing was 14.8, which is greater than five. Thus, the sample size was deemed sufficient for the model [31].

## 3. Results

A total of 532 individuals living with HIV (305 with HIV and 227 with HIV and LD) were included in the study. There were no significant differences between the groups with regard to gender, race, employment status, and household income. However, subjects with HIV and LD were significantly older and less educated than the HIV group without LD (Table 1). Participants had a mean CD4 count of 455 cells/mm^3^ (range 44–1540 cells/mm^3^) and 59% of the overall sample had an undetectable HIV viral load (Table 2). The classifications of types of comorbid LD are noted in Table 3. All others without evidence of LD were classified as HIV. 

The baseline hypothesized Wilson and Cleary model was assessed with SEM and found to hold true in both groups, persons living with HIV without LD and in persons living with HIV and LD, when a Lagrange multiplier modification was applied. The baseline model parameters and subsequent model modifications are noted in Table 4. Model 1 parameters were found after releasing model constraints and allowing additional pathways from symptom status to general health perceptions and overall quality of life, and from biological/physiological factors to general health perceptions. Model 2 allowed for correlations between the biological/physiological factors (CD4 count and HIV viral load) and the error terms. There were no significant differences in the HIV group model and the HIV and LD model. A multisample robust SEM was performed. Because there were no significant differences between the structural models in the two groups the data were constrained to fit the same model. The constrained model estimates one set of parameters for both groups. SEM was run on both the constrained and unconstrained models and it was found that the constrained model had a better fit. The model parameters were not significantly different when comparing the Satorra-Bentler model chi-squares.

There was a significant direct effect of biological/physiological factors on symptom status, as measured with the MOS-HIV mental summary score, in persons with HIV and persons with HIV and LD. CD4 count, as a measure of biological/physiological factors, significantly predicted symptom status, as measured with the MOS-HIV mental summary score in both the HIV (*B* = .120, *z* = 2.44, *P* = .015) and HIV and LD groups (*B* = .113, *z* = 2.44, *P* = .015). HIV viral load as a measure of biological/physiological factors significantly predicted symptom status, as measured with the MOS-HIV mental summary score in both the HIV (*B* = .103, *z* = 2.10, *P* = .036) and HIV and LD groups (*B* = .107, *z* = 2.10, *P* = .036). An additional significant path was identified using the Lagrange multiplier from biological/physiological factors to general health perception (CD4 count to perception of illness) in both the HIV group (*B* = .122, *z* = 2.97, *P* = .003) and the HIV and LD group (*B* = .103, *z* = 2.97, *P* = .003). CD4 count and HIV viral load, as measures of biological/physiological factors, did not predict symptom status, as measured by the BDI-II.

There was a significant direct effect of symptom status on functional status in both groups. However, categorized missed appointments (“yes” or “no”) was not related to the study measures and was therefore not included in the SEM/path analysis. The MOS-HIV mental summary score, as a measure of symptom status, significantly predicted functional status, as measured with the MOS-HIV physical summary score, in both the HIV (*B* = .669, *z* = 11.52, *P* < .001) and HIV and LD groups (*B* = .644, *z* = 11.52, *P* < .001). The BDI-II, as a measure of symptom status, did not predict functional status, as measured by the MOS-HIV physical summary score.

There were four significant additional paths identified by using the Lagrange multiplier linking symptom status, as measured by the BDI-II and the MOS-HIV mental summary score, to distal components of the Wilson and Cleary model of HRQOL. Three of the identified paths linked symptom status directly to general health perceptions and one linked symptom status directly to overall QOL. The MOS-HIV mental summary score, as a measure of symptom status, significantly predicted general health perceptions, as measured with the perception of illness visual analogue scale in both the HIV (*B* = .410, *z* = 4.63, *P* < .001) and HIV and LD groups (*B* = .369, *z* = 4.63, *P* < .001). The MOS-HIV mental summary score, as a measure of symptom status, significantly predicted general health perceptions, as measured with the MOS-HIV health transition score, in both the HIV (*B* = .343, *z* = 4.12, *P* < .001) and HIV and LD groups (*B* = .319, *z* = 4.12, *P* < .001). The BDI-II, as a measure of symptom status, significantly predicted general health perceptions, as measured with the MOS-HIV health transition score in both the HIV (*B* = −.204, *z* = −2.94, *P* = .003) and HIV and LD groups (*B* = −.203, *z* = −2.94, *P* = .003). The final added path included the BDI-II, as a measure of symptom status, which significantly predicted overall QOL, as measured with the satisfaction with life scale, in both the HIV (*B* = .387, *z* = −6.84, *P* < .001) and HIV and LD groups (*B* = .358, *z* = −6.84, *P* < .001).

There was no significant direct effect of functional status, as measured with the MOS-HIV physical summary score, on general health perceptions, as measured with either the perception of illness visual analogue score or the MOS-HIV health transition score, in persons with HIV without liver disease or in persons living with HIV and LD.

**Figure 3:**
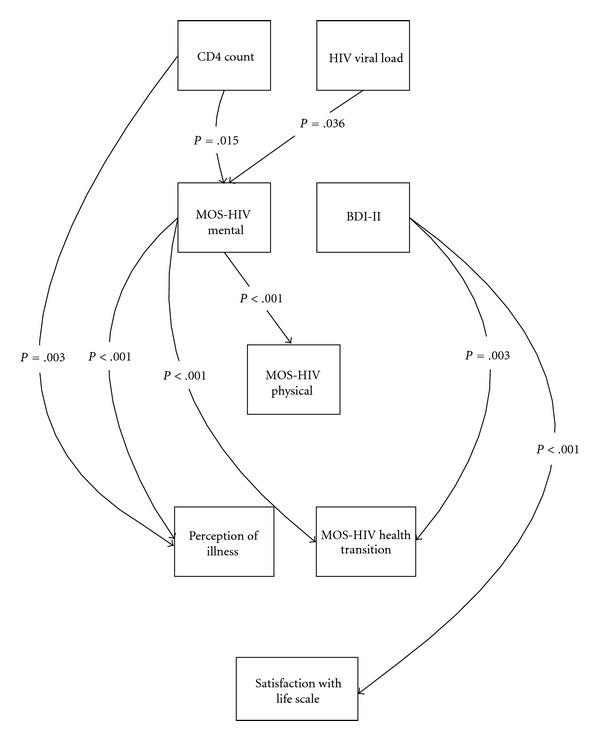
SEM with significant measured modeled pathways retained (*n* = 532).

There was no significant direct effect of general health perceptions, as measured by either the perception of illness visual analogue score or the MOS-HIV health transition score, on overall QOL, as measured by the satisfaction with life scale, in either group. The significant retained SEM modeled pathways are depicted in Figure 3.

Of the 36 covariance pathways that were added to the good-fitting model, 11 individual parameters were found to be significant. There were four significant pathways found stemming from the characteristics of the individual. Three of the four came from race/ethnicity and the remaining path related age to functional status. The covariate of age predicted the endogenous variable of functional status, as measured by the MOS-HIV physical summary score (*B* = .069, *z* = −1.984, *P* = .047). Age was not related to measures of symptom status, general health perceptions, or overall QOL. Race as a covariate in the model was found to have three independent significant paths. The first path that race predicted was symptom status, as measured by the MOS-HIV mental summary score (*B* = .091, *z* = 2.204, *P* = .028). The second path predicted by race was functional status, as measured by the MOS-HIV physical summary score (*B* = .125, *z* = 3.350, *P* = .001). The last path predicted by race was general health perceptions, as measured by the MOS-HIV health transition score (*B* = .148, *z* = 3.443, *P* = .001). Sex and years of education were not significantly related to the main Wilson and Cleary model components (biological/physiological factors, symptom status, functional status, general health perceptions, and overall QOL). 

**Figure 4:**
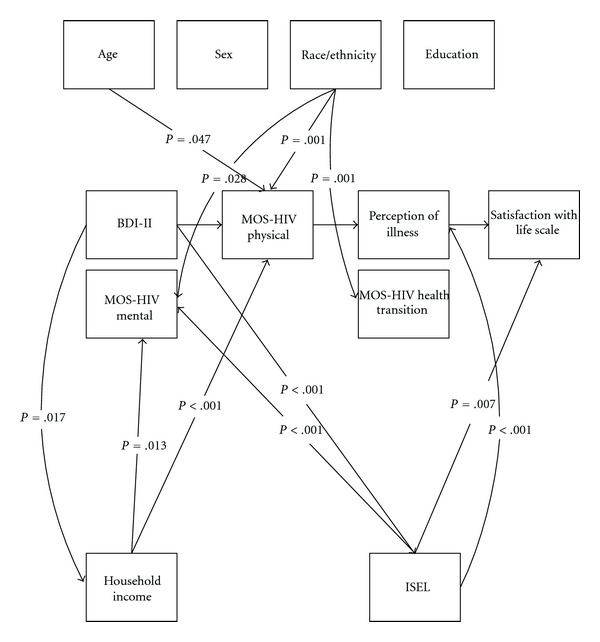
SEM with significant covariate model pathways retained.

The characteristics of the environment as measured by the ISEL (social support) and household income had significant independent covariant relationships to the model parameters. Four paths linked from social support and three paths linked from gross annual household income. Specifically, self-reported social support, as measured with the ISEL, had an independent effect on symptom status at a significance level of <.001 as measured by both the BDI-II (*B* = .517, *z* = −11.936, *P* < .001) and MOS-HIV mental summary score (*B* = .525, *z* = 11.685, *P* < .001). The ISEL score as a covariate in the model also independently predicted general health perceptions, as measured by the perception of illness visual analogue scale (*B* = .228, *z* = 4.180, *P* < .001). Lastly, overall QOL, as measured by the satisfaction with life scale, was independently predicted by the ISEL (*B* = .155, *z* = 2.707, *P* = .007). Gross annual household income, as a measure of characteristics of the environment, had three significant independent paths. First, gross annual household income as a covariate in the model predicted symptom status by both the BDI-II (*B* = .104, *z* = −2.392, *P* = .017) and the MOS-HIV mental summary score (*B* = .113, *z* = 2.488, *P* = .013). Gross annual household income also independently predicted functional status, as measured by the MOS-HIV physical summary score (*B* = .165, *z* = 4.024, *P* < .001). The significant retained independent multigroup SEM covariate pathways are depicted in Figure 4.

## 4. Discussion

This study applied a sophisticated statistical analysis, SEM, to test a theoretical model of HRQOL as described by Wilson and Cleary. The assessment of HRQOL is useful not only for capturing important facets of a person's self-perception of how illness affects daily functioning, but also as a valid measure of clinical outcome when assessing interventions. This model, as initially conceptualized by Wilson and Cleary, has been found useful to describe HRQOL in persons living with HIV. However, persons living with HIV have an increased risk of developing liver disease as related to toxic effects of antiretroviral therapy yielding hepatitis and other liver disease. These findings are similar to other studies that have used the Wilson and Cleary model in clinical samples with heart failure, gastrointestinal bleeding, diabetes, and Hodgkin's lymphoma. Therefore, the model proposed by Wilson and Cleary has now been supported in a sample of individuals with HIV and LD.

The primary aim of the study was to test the fit of the Wilson and Cleary model of HRQOL in two groups of patients, persons living with HIV without LD and persons living with HIV and LD. Wilson and Cleary's model was found to be fit for both groups.

The secondary aim was to test if each variable was directly linked as hypothesized by Wilson and Cleary. In both groups, the biological/physiologic factors (CD4 count and HIV viral load) had a significant direct effect on symptom status, which in turn had a significant direct effect on functional status. The additional pathways between symptom status and other components, such as QOL, have been noted by other researchers and suggest that self-reported depressive symptoms and mental function are important indicators of HRQOL [32–42].

Other possible factors influencing HRQOL, such as characteristics of the individual and of the environment, were investigated. Additionally, 36 parameters were assessed simultaneously. Social support was associated with both general health perceptions and overall QOL. Social support was also significantly related to mental symptom status. Total gross annual household income was related to MOS-HIV mental and physical summary scores. Thus, characteristics of the environment, as others have shown, have relevance to the components of HRQOL [43–45].

There are several limitations in this study. First, a longitudinal study would be necessary with data gathered from different time points to assess the causal relationships. The data used in this study were cross-sectional and therefore the causal relationships were not able to be assessed. Second, the higher proportion of non-white participants and lower income levels, or specific populations such as homeless and injection-drug users, may aid in making this study more generalizable as these differences are more reflective of the overall U.S. population of persons living with HIV. In addition, not having access to a telephone may have further biased the sample recruited. 

## 5. Conclusions

The findings of this research show that both health-related factors, such as CD4 count and HIV viral load, and social factors, such as self-reported mental health and depressive symptoms, are important indicators of HRQOL in this sample of persons living with HIV (*N* = 532). These findings imply that symptom status, specifically depressive symptoms and altered mental function, is a key issue in determining HRQOL in persons with HIV without LD and in persons with HIV and LD.

There is no cure for HIV. Therefore, persons living with HIV with or without LD are no longer just trying to survive day to day; rather, they are seeing the future of living with HIV as a chronic disease with debilitating long-term consequences. Direction for a future study needs to include better measures of biological/physiological factors that specifically assess current disease status. Specifically, work needs to focus on a specific liver disease that may significantly impact persons living with HIV such as the hepatitis C virus or on specific symptoms that affect the individual most, such as depressive symptoms.

Depressive and mental symptoms had the strongest relationship to the other model measures suggesting that a focus for clinical intervention would be to more closely address these issues in persons living with HIV and LD. These findings also suggest that a more complete understanding of the symptom experience in persons living with HIV and LD is fundamental to achieve optimal patient outcomes. It may be that specific symptoms, such as depressive symptoms, need to be controlled for model testing. Future research should consider a symptom-specific tool to look for clusters of symptoms in both HIV and liver disease. This study also found that race, social support, and income were important covariates. Therefore, another direction for future research would be to assess differences between racial and socioeconomic status on HRQOL in persons with HIV and LD.

## Figures and Tables

**Table 1:** Characteristics of demographics in HIV and HIV + LD groups.

Variable	Overall (*N* = 532)	Group		Statistic		
		HIV (*N* = 305)	HIV + LD (*N* = 227)			
	*n* (%)/*M* (SD)	*n* (%)/*M* (SD)	*n* (%)/*M* (SD)	Chi-sq/*t*-test		*P* value
				*df *	Value
Sex				1	1.63	.201
Male	371 (69.7)	206 (67.5)	165 (72.7)
Female	161 (30.3)	99 (32.5)	62 (27.3)
Race				1	1.79	.181
White	261 (49.1)	142 (46.6)	119 (52.4)
Non-white	271 (50.9)	163 (53.4)	108 (47.6)
Total gross annual household income				5	8.08	.152
Under 10 000	262 (49.2)	140 (45.9)	122 (52.9)
10 000 to 13 000	90 (16.9)	53 (17.4)	37 (16.3)
13 000 to 20 000	60 (11.3)	35 (11.5)	25 (11.0)
20 000 to 30 000	39 (7.3)	29 (9.5)	10 (4.4)
30 000 to 50 000	34 (6.4)	23 (7.5)	11 (4.8)
Over 50 000	32 (6.0)	17 (5.6)	15 (6.6)
Missing	15 (2.8)	8 (2.6)	7 (3.1)
Age	42.40 (7.86)	41.51 (8.29)	43.87 (7.14)	530	−3.44	.001
Number of years of education	13.21 (2.76)	13.35 (2.83)	12.77 (2.66)	529	2.37	.018

**Table 2:** Measure comparisons by model variable for HIV and HIV + LD groups.

Variable measure	Overall (*n* = 532)	Group		Statistic		
		HIV (*n* = 305)	HIV + LD (*n* = 227)			
	*n* (%)/*M* (SD)	*n* (%)/*M* (SD)	*n* (%)/*M* (SD)	Chi-sq/*t*-test		
				*df *	Value	*P* value
Biological/physiological factors						
CD4 count	455.94 (303.97)	492.25 (341.98)	435.43 (316.30)	Mann-Whitney	−2.01	.044
HIV viral load				Pearson (1)	3.35	.067
Detectable	199 (41.1)	102 (37.5)	97 (45.8)			
Undetectable	285 (58.9)	170 (62.5)	115 (54.2)			
Symptom status						
Beck depression inventory-II	14.94 (11.54)	13.89 (11.68)	16.34 (11.22)	525	−2.42	.016
MOS-HIV mental summary score	45.44 (12.06)	46.77 (12.24)	43.63 (11.59)	520	2.96	.003
Functional status						
Missed appointments				Pearson (1)	.835	.361
Yes	172 (33.0)	94 (31.3)	78 (35.1)			
No	350 (67.0)	206 (68.7)	144 (64.9)			
MOS-HIV physical summary score	41.80 (11.62)	43.47 (11.28)	39.51 (11.72)	520	3.90	<.001
Perception of illness	0.74 (0.19)	.74 (.18)	.731 (.19)	524	.72	.470
MOS-HIV health transition score	3.34 (1.02)	3.39 (1.01)	3.27 (1.02)	524	1.34	.182
Overall quality of life						
Satisfaction with life scale	3.57 (1.49)	3.63 (1.44)	3.49 (1.56)	517	1.08	.280

**Table 3:** Classification of comorbid types of liver disease (*n* = 227).

HIV and type of liver disease	*N*	Cumulative total	Percentage (%)
HIV + Hepatitis A only	15	15	6.6
HIV + Hepatitis B only	32	47	14.1
HIV + Hepatitis C only	88	135	38.8
HIV + Hepatitis A and B	7	142	3.1
HIV + Hepatitis A and C	1	143	0.4
HIV + Hepatitis B and C	13	156	5.7
HIV + Hepatitis A, B and C	5	161	2.2
HIV + Unknown Hepatitis	52	213	22.9
HIV + Other liver disease	12	225	5.3
HIV + Other liver disease + Hepatitis C	1	226	0.4
HIV + Other liver disease + Hepatitis A, B, and C	1	227	0.4

**Table 4:** Goodness of fit summary for model selection.

	SB *χ* ^2^	*df*	CFI	RMSEA	Δ SB *χ* ^2^	*df*	*P* value
Baseline	22.68	20	.997	.028	—	—	—
Model 1 regression coefficent constrained	39.31	36	.997	.020	16.61	16	.411
Model 2 regression coefficent constraints with parsimony	40.31	38	.998	.017	17.57	18	.484

Note: SB *χ* ^2^: Satorra-Bentler scaled chi-square; *df*: degrees of freedom; CFI: comparative fit index; RMSEA: root mean squared error of approximation; Δ: difference.
